# Development of sustainable downstream processing for nutritional oil production

**DOI:** 10.3389/fbioe.2023.1227889

**Published:** 2023-10-10

**Authors:** Samuel Rollin, Adarsha Gupta, Christopher M. M. Franco, Surinder Singh, Munish Puri

**Affiliations:** ^1^ Medical Biotechnology, College of Medicine and Public Health, Flinders University, Adelaide, SA, Australia; ^2^ Flinders Health and Medical Research Institute, College of Medicine and Public Health, Flinders University, Adelaide, SA, Australia; ^3^ Nourish Ingredients, Canberra, NSW, Australia

**Keywords:** cell disruption, green solvents, DHA, microalgae, nanotechnology, nutrition, omega-3 fatty acids, thraustochytrid

## Abstract

Nutritional oils (mainly omega-3 fatty acids) are receiving increased attention as critical supplementary compounds for the improvement and maintenance of human health and wellbeing. However, the predominant sources of these oils have historically shown numerous limitations relating to desirability and sustainability; hence the crucial focus is now on developing smarter, greener, and more environmentally favourable alternatives. This study was undertaken to consider and assess the numerous prevailing and emerging techniques implicated across the stages of fatty acid downstream processing. A structured and critical comparison of the major classes of disruption methodology (physical, chemical, thermal, and biological) is presented, with discussion and consideration of the viability of new extraction techniques. Owing to a greater desire for sustainable industrial practices, and a desperate need to make nutritional oils more available; great emphasis has been placed on the discovery and adoption of highly sought-after ‘green’ alternatives, which demonstrate improved efficiency and reduced toxicity compared to conventional practices. Based on these findings, this review also advocates new forays into application of novel nanomaterials in fatty acid separation to improve the sustainability of nutritional oil downstream processing. In summary, this review provides a detailed overview of the current and developing landscape of nutritional oil; and concludes that adoption and refinement of these sustainable alternatives could promptly allow for development of a more complete ‘green’ process for nutritional oil extraction; allowing us to better meet worldwide needs without costing the environment.

## 1 Introduction

Nutritional oil is defined as an oil possessing essential fatty acids and unsaturated fatty acids beneficial to the establishment and maintenance of human health and wellbeing (from paediatric to geriatric). These oils are derived from external dietary sources, thereby leading to the varying levels among the general population. Nutritional oils are considered vital to continued maintenance of homeostasis, and valuable for improving general health with examples, including linoleic acid (LA, C18:2), arachidonic acid (ARA, C18:4n6) and palmitoleic acid (PTA, C16:1) (Di Pasquale, 2009). Single-cell oils (SCO), microbial oils produced by unicellular organisms such as microalgae and yeasts, may thus be classified as nutritional oil based upon their fatty acid profile (Armenta and Valentine, 2013). However, not all nutritional oils are equally able to promote positive nutritional status, making some more highly sought after than others, in particular omega-3 nutritional oils are considered a valuable nutritional oil class (Di Pasquale, 2009). Polyunsaturated fatty acids (PUFAs) (existing alongside saturated fatty acid/SFAs and monounsaturated fatty acids/MUFAs) are a major class of long-chain fatty acids, which comprise the majority of all edible nutritional oils; being chemically defined by having three or more unsaturated carbons (D’Alessandro and Antoniosi Filho, 2016). Docosahexaenoic acid (DHA), together with eicosapentaenoic acid (EPA), and docosapentaenoic acid (DPA) are a part of the omega-3 (ω-3) class of PUFAs, which are often cited as highly nutritional due to frequent links observed between high ω-3 levels, cognitive function, visual function, joint mobility and pain reduction in arthritis, and cardiovascular health (Kaur et al., 2011; Cleland and James, 2012; Swanson et al., 2012; Nicholson et al., 2013; Ghasemi Fard et al., 2019; Sinclair, 2019; DiNicolantonio and O'Keefe, 2020; Katiyar and Arora, 2020). As a result, both DHA and EPA are among the most highly valued nutritional oils and incorporation of a large proportion into the diet is highly desirable.

SCO are rapidly growing in value as nutraceuticals to supplement often insufficient dietary levels; especially in Eurasia and the United States (Orozco Colonia et al., 2020; Ferreira de Oliveira and Bragotto, 2022). As the century advances, it becomes increasingly challenging to ensure that a growing global population receives all its essential nutrients (Torres-Tiji et al., 2020; Chen et al., 2022). As a result, companies set-up to produce biofuels are increasingly switching towards the production of nutritional oils due to their improved economic feasibility compared to biocrude oils (Maeda et al., 2018). This is in part due to inherent difficulties in biofuel production which currently make biorefineries struggle to compete with crude oil refineries; although there is scope for this to be solved in the future (Severo et al., 2019; Olguin et al., 2022). The worldwide nutraceutical market in 2010 was estimated at $1.69 billion USD; in which omega-3 supplements were valued at $34000–112000 USD per tonne with an estimated market size of $61884 USD per year (not including alternative uses, such as inclusion in ω-3 fortified foods) (Laurens et al., 2017). In 2018, the value of PUFAs, such as ω-3 FAs was estimated between $35437 and $88593 USD per tonne (Barsanti and Gualtieri, 2018). This is highly significant given estimates that for all humans to meet the recommended daily ω-3 intake 1.3 million tonnes would be required to be produced annually (Finco et al., 2017). More recent estimates expect the value of the ω-3 FA market alone to rise to $58.76 billion USD by 2025 (6% annual growth rate, 2020–2025) (Orozco Colonia et al., 2020). Currently, most commercially available ω-3 supplements are produced using fish-extracted oils, which is an issue for many reasons including taste, smell, and availability to vegetarians/vegans; yet the most concerning issue is sustainability due to continuously declining fish stocks and the consistent need for adequate dietary ω-3 (Mishra et al., 1993; Cuellar-Bermudez et al., 2015). To meet the worldwide demand many alternative ω-3 sources have been considered including, yeasts, GMO plants, fungi and oleaginous microalgae species such as *Scenedesmus, Haematococcus,* and *Chlorella* with a highly promising principal source being microalgal ‘Thraustochytrids’ (Puri et al., 2013; Matos, 2017; Fossier Marchan et al., 2018). Microalgae are considered the most promising source for new food products as in addition to their PUFA content they’re also rich in proteins, pigments, vitamins, and other bioactives (Chen et al., 2022; Janssen et al., 2022). Oleaginous microalgae are particularly popular due to their significantly higher yield when compared to fish and plant-based alternatives (Finco et al., 2017). While there remain many challenges in exploiting microalgae-based ω-3 fatty acids, the increasing requirement for new solutions has led to certain countries and institutions pursuing the necessary steps to ensure the viability of these microalgal-processes (Tocher et al., 2019; Magoni et al., 2022). Indeed the number of patents worldwide for the production of ω-3 PUFAs from microalgae, such as Thraustochytrids has been steadily rising from 2000–2023 (a total of 658 patent applications in this period); with many institutions poised to accelerate production once economic and environmental feasibility of downstream processing are finally achieved (Orozco Colonia et al., 2020).

Thraustochytrids are a grouping of unicellular (single-celled) heterotrophic protists (approx. 6–21 μm in diameter) in the *‘Labyrinthulomycetes’* class of the *‘Heterokonta’* phylum, which are of particular interest due to their significant bioactive-producing capabilities (Gupta et al., 2012; Lee Chang et al., 2012; Fossier Marchan et al., 2018; Morabito et al., 2019). While Thraustochytrids produce many valuable products, such as pigments, minerals, vitamins and bioactive peptides, they are best known for their high proportion of cellular lipids, which often comprise over 50% of dry cell weight (Raghukumar, 2008; Santos-Sánchez et al., 2016; Menegazzo and Fonseca, 2019; Tran et al., 2020). Fatty acids (FAs) are the major component of these lipids that are among the most highly valued microalgal products (Cuellar-Bermudez et al., 2015; Xue et al., 2018). A large proportion of these FAs (many of which exist in cytoplasmic micelles) are long-chain PUFAs, with DHA often comprising 25% of all Thraustochytrid lipids, going up to 30%–40% of lipids in rare cases (Gupta et al., 2012; Lenihan-Geels et al., 2013; Li et al., 2019). This is likely because a high proportion of unsaturated FAs allows higher lipid fluidity at low temperatures which is beneficial for these marine microorganisms as they must retain cell fluidity to function and survive (Shene et al., 2020). This high FA proportion coupled with the greater degree of control over microalga cultivation compared to fish (reducing the accumulation of pollutants and toxins in derived oil) and positive reception by key consumer demographics has resulted in several companies looking to utilise the thraustochytrids for the production of value-added products (Orozco Colonia et al., 2020).

Hence, the potential to extract SCOs (such as ω-3) from Thraustochytrids efficiently is a critical step. The ability of microalgae to grow in controlled conditions is beneficial for optimisation and upscaling, with extensive studies conducted on Thraustochytrid lipid production and growth strategies. (Cuellar-Bermudez et al., 2015; Leone et al., 2019). Currently, downstream processing (FA extraction/purification) accounts for 50%–80% of all production costs associated with separating desired ω-3 fatty acids; and frequently used methods are often time-consuming, energy-consuming and/or polluting due to the toxic nature of the required solvents (i.e., hexane, chloroform) (Kumari et al., 2011; Li et al., 2019; Sinclair, 2019). A major issue regarding the use of toxic solvents in extracting lipids is the risk associated with products intended for human consumption where solvents are incompletely removed. These toxic solvents pose a hazard to the producer, consumer, and the environment and are undesirable for long-term usage in industrial production (Kumari et al., 2011; Li et al., 2019). Together with the high energy consumption for many pre-treatments, the use of solvents generally has an unfavourable environmental impact, which is not justified by the result due to frequently slow or incomplete FA recovery. Extraction is thus considered to be a major ‘bottleneck’ in the commercialisation of microalgae-derived oils and their wider use (Enamala et al., 2018; Gifuni et al., 2019). Hence, the development and optimisation of methods utilising ‘greener’ solvents and more environment-friendly processes is a valuable prospect and could serve to make extraction and purification of ω-3s, such as DHA from Thraustochytrids not only more efficient and affordable (lower production losses), but also more environmentally viable in the long-term.

Many of the procedures required to separate these nutritious oils from various microbial sources (*Mortierella* sp.) are inefficient, energy-consuming or produce toxic waste/by-products which is highly unattractive for commercial exploitation. Hence the development of ‘greener’ eco-friendly practices is essential for continued growth of the nutritional oil industry.

In this review, we considered four major areas in fatty acid downstream processing (extraction, cell disruption, solvents and purification) that pose a bottleneck in the wider exploitation of oleaginous microalgae for nutraceutical purposes and propose more sustainable alternatives in comparison to existing procedures. Additional consideration of applying existing nanomaterials to fatty acid separation to improve the sustainability of nutritional oils through innovative downstream processing is discussed.

## 2 Fatty acids as single cell oils (Omega-3 FAs and MUFAs)

### 2.1 Current sources of Omega-3 fatty acids

Due to lack of ω-3 synthesis in humans it is essential to gain ω-3 FAs from dietary sources (Lewis et al., 2000). It is recommended that men and women receive 1.6 g and 1.1 g of dietary ω-3 daily, which should contain a minimum of 10% DHA (Gogus and Smith, 2010; Nicholson et al., 2013). This tallies with the recommendations of Australia’s National Health and Medical Research Council (the NHMRC), who suggest at minimum adult males take 1.46 g of ω-3 daily (of which 160 mg must be long chain ω-3, such as DHA/EPA) and adult females take 0.89 g of ω-3 daily (of which 90 mg must be long chain ω-3 such as DHA/EPA). (NHMRC, 2014). Essential ω-3s, such as DHA and EPA, are lacking in terrestrial plants and crops, hence, it is imperative to explore alternative sources (Katiyar and Arora, 2020). Several studies have reported on GM production of ω-3 in staple crops (Zhou et al., 2023). By far, the largest natural dietary ω-3 source is fish, yet most individuals do not eat sufficient fish to meet daily needs. Thus, ω-3 oil is commonly extracted from fish for use as a dietary supplement (Eratte et al., 2018). Unfortunately, fish oils are renowned for unpleasant taste/smell, mainly due to the compounds present along with the ω-3 (toxic compounds, which may cause harm over long periods, such as methylmercury, or dioxins that have been found in fish oils) (Byreddy et al., 2015; Eratte et al., 2018; Verma et al., 2019). Fish oils are unsuitable to vegetarian or vegan diets due to their animal tissue origin, and can be inconsistent, with differences in quality based on season and location (Cuellar-Bermudez et al., 2015; Katiyar and Arora, 2020). As a source, fish are highly unsustainable due to rapid stock depletion, and thus are not a reasonable avenue to produce sufficient oil for the rising ω-3 FA demand (Puri, 2017; Orozco Colonia et al., 2020). Hence, microalgal-derived single-cell oil has the potential to be a strong sustainable alternative to fish oil and transgenic plants alongside its use as an important renewable biofuel (Kukreja et al., 2017).

### 2.2 Structure and function of Omega-3 fatty acids

Chemically, ω-3 fatty acids are defined by a double-bond between the third and fourth carbon from the terminal methyl groups (Gupta et al., 2012; Cuellar-Bermudez et al., 2015). In particular, the molecular chain of DHA is 22 carbons in length, containing 6 cis-double bonds (22:6), and has a methyl group at one end (as per the ω-3 class) with a carboxylic acid group at the other end (as per the FA class) (Gupta et al., 2012). This high level of unsaturated carbons often pose an issue in extraction due to undesired auto-oxidation and is a major factor in the inability of the human body to synthesise its own ω-3 FAs due to the lack of enzymes required to remove hydrogens from precursor compounds (Leone et al., 2019; Li et al., 2019). This is a significant concern as previous studies have found that an imbalance in ω-6 (common in plants) relative to ω-3 may play a role in the incidence of carcinogenesis, with a ω-6 to ω-3 ratio of 1:1 highly desirable (Hooper et al., 2006; Nicholson et al., 2013; Morabito et al., 2019; Katiyar and Arora, 2020). Furthermore, ω-3 is essential for foetal eye and brain development and continues to be vital to sight and cognitive processes in adults, as well as benefitting cardiovascular function (Armenta et al., 2009; Gogus and Smith, 2010; Lee Chang et al., 2012; Xue et al., 2018). Metabolites produced from DHA, DPA and EPA in the body may also have a downregulatory effect reducing circulating inflammatory compounds (Nicholson et al., 2013; Markworth et al., 2016; Santos-Sánchez et al., 2016). Hence ω-3 FAs are considered essential in reducing the impact of non-communicable chronic disease (Katiyar and Arora, 2020). In accordance with this established ‘essentiality’ we now aim to present a comprehensive review of extraction processes, solvents, and pre-treatment strategies valuable for more sustainable ω-3 FA production.

### 2.3 Monounsaturated fatty acids (MUFAs)

Monounsaturated fatty acids are the fatty acids that contain only one double bond in their structure, providing them specific properties in relation to their interactions with other fatty acids in the human body (Jenkins et al., 2020). There MUFAs include fatty acids, such as palmitoleic, oleic, elaidic and vaccenic acids and are abundantly found in various foods, including nuts and plant oils (e.g., olive oil). MUFAs-rich diets have demonstrated favorable anti-inflammatory and cardiovascular benefits, with improved lipid profile (Cheah et al., 2019). Along with PUFAs, Thraustochytrids can also be a source of MUFAs as the fatty acid profiles of these strains have shown the presence of palmitoleic acid, oleic acid and vaccenic acid, which can be manipulated to produce in high proportion using various fermentation conditions. However, this study focused on the PUFAs-rich lipids extraction process.

### 2.4 The complexity of cell wall for lipid release

A key problem in developing a universal ω-3 PUFA extraction process from microalgae is due to differences in the cell structure and FA composition (Kumari et al., 2011; Byreddy et al., 2015). For example, thraustochytrids are single-celled unicellular organisms, usually containing multiple ‘free’ lipid bodies in the cytoplasm (Jain et al., 2005; Fossier Marchan et al., 2018). All members of the *Thraustochytrium* genus have a non-cellulosic wall that is unique compared to the typical cellulose walls of other microalgae or chitin walls of fungal cells (Fossier Marchan et al., 2018). This cell wall is composed of 2–3 nm thick circular scales which cover the surface of the cell membrane. These scales consist of sulphated polysaccharides, containing a high proportion of galactose and xylose (Chamberlain and Moss, 1988; Jain et al., 2005). Other carbohydrates such as mannose, glucose, rhamnose and 3-O-Me-Galactose have also been observed across different Thraustochytrids (Fossier Marchan et al., 2018). Additionally, one study reported the detection of high calcium levels within these scales, with the presence of silica also observed in certain species (Chamberlain and Moss, 1988). In addition to the thraustochytrid strains, other microalgae species, such as *Chlorella* sp., *Scenedesmus* sp., *Nannochloropsis* sp. have also been studied for enhancing several bioproducts recovery from the cells, including lipids (Rahman et al., 2022; Weber et al., 2022). For example, the cell wall composition of *Chlorella* sp. consists of polysaccharides (rigid structures), sugars, proteins and uronic acids making it difficult to disrupt the cells to release bioproducts, thus, Weber and co-workers (2022) investigated the stepwise cell wall disintegration of *Chlorella vulgaris* using various chemicals and mechanical processes to enhance the bioproducts recovery. Thus, it was ascertained that since there are limited studies about the most appropriate method for these types of specific cell wall composition, it is necessary to consider all existing possible treatments and procedures for efficient FA recovery.

## 3 Extraction processes

### 3.1 An outline of standard downstream processes

**FIGURE 1:**
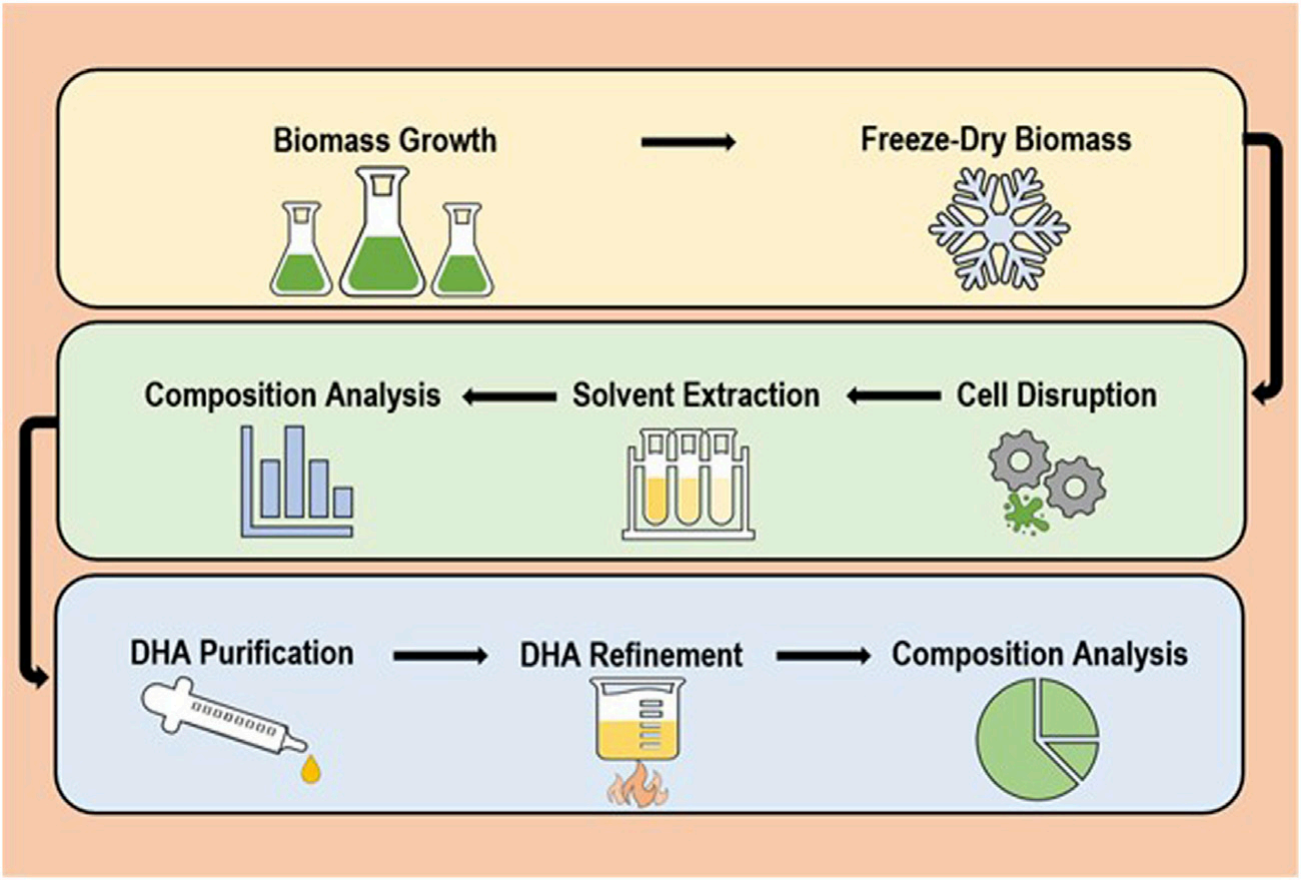
General overview of the standard lipid recovery process.

The complete process of ω-3 oil production can be generalised to a sequence of six major steps (and two quality checks) which are as follows: biomass growth > biomass drying > cell disruption > solvent extraction > composition analysis 1 > PUFAs purification > PUFAs refinement > composition analysis 2 (Gunstone, 1996; Adarme-Vega et al., 2012; Cuellar-Bermudez et al., 2015; Meullemiestre et al., 2015). A visual representation is demonstrated in Figure 1. Among these, stages requiring major development/optimisation are cell disruption, solvent extraction and DHA purification; mainly due to a reliance on energy consuming/expensive mechanical procedures, use of toxic solvents and product loss between steps. For each of these multiple interchangeable methods are available that can be tailored and combined for achieving the best efficiency and end-product. However, it is highly recommended that oil products for human consumption are extracted with non-toxics to reduce unnecessary risks (Kumari et al., 2011; Li et al., 2019). In addition, it is advised that extraction is performed under anaerobic conditions to avoid PUFAs auto-oxidation (Li et al., 2019). The 4-step refining process (from degumming to deodorisation) is well established and thus not a major focus of this review (Gunstone, 1996).

This overall method differs slightly based on whether cells are freeze-dried (dry biomass) or in solution (wet biomass), with no drying process required for wet biomass (Kumar and Singh, 2019; Li et al., 2019). Nevertheless, biomass type influences the type of methods that can be subsequently used for each stage as solvent extraction is most effective with minimal water present (Dibendetto, 2012; Howlader et al., 2018). Lipid recovery from wet biomass is frequently limited by lipid accessibility, mass transfer and emulsion formation, which can be easily addressed by improved cell disruption techniques and better solvents (Dong et al., 2016; Harris et al., 2018). It is vital to develop improved wet algae FA extraction as the most commonly used freeze-drying process is costly and energy consuming (Cho et al., 2013; Howlader et al., 2018). Typically, there are three types of extraction processes, destructive, semi-destructive or non-destructive (Dibendetto, 2012). Due to the prerequisite of a cell disruption step to achieve best possible yield, destructive processes will be the focus. While it is essential to develop extraction methods with a ‘green’ focus; processes must be scalable and affordable (Adarme-Vega et al., 2012; Koyande et al., 2019; Li et al., 2019).

**FIGURE 2:**
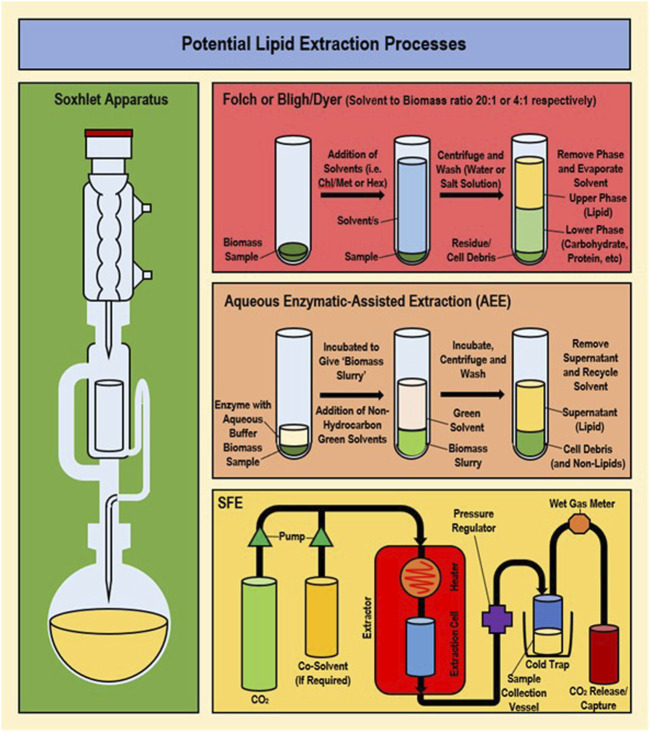
Overview of methodology/apparatus set-up for traditional and non-traditional extraction processes (Folch/Bligh-Dyer, Soxhlet, AEE and SFE).

While cell disruption methods and solvent types are frequently replaced or modified more than the solvent extraction method (which generally follows a standard procedure), successful modifications have been achieved previously and newer methods such as supercritical fluid extraction (SFE) and aqueous enzymatic extraction (AEE) are increasingly available for exploitation (Patil et al., 2018; Anto et al., 2020; Liu et al., 2020). These newer methods are comparatively lower in toxicity and energy requirements than traditional solvent extraction procedures. In addition, methods involving simultaneous cell disruption and extraction are available including microwave-assisted extraction (MAE), ultrasound-assisted extraction (UAE) and surfactant-assisted extraction (SAE). However, each part (i.e., disruption or extraction) is discussed separately in this study due to ‘issues associated with their disruption component that make them less suitable for an environment-friendly methodology (while the paired extraction is generally the typical solvent extraction method with solvents interchanged). (Harris et al., 2018). A comparison of the standard solvent extraction methods along with newer ‘green’ methods is given in Supplementary Table S1A, and major methods are provided in Figure 2.

### 3.2 Green extraction procedures

Extraction methods that have been frequently used include direct transesterification, saponification, aqueous enzymatic extraction (AEE) and supercritical fluid extraction (SFE). Unfortunately, saponification is unsuitable for PUFAs due to low yields; while trans-esterification allows a greater FA yield than normal extraction, yet conversion of esterified products back to FAs is energy consuming (involves additional steps), making wider use undesirable (Lewis et al., 2000; Li et al., 2019). AEE (otherwise known as enzyme-assisted aqueous extraction processing or EAEP) utilises different enzyme compositions to extract lipids out of cell biomass using water as the solvent for oil extraction (Liang et al., 2012; Du et al., 2017; Kumar et al., 2017; Liu et al., 2020). AEE is highly environment-friendly, safe and more cost effective than conventional extraction (Wu et al., 2017). However, temperature and pH must be tightly controlled for maintaining the enzyme activity, and AEE often forms an emulsion which is limiting due to the requirement of adding a demulsification step (Kumar et al., 2017). Use of microwave demulsification may address this and has previously improved oil quality (Liu et al., 2020). Multiple disruption methods have been combined with AEE achieving relative success, such as the combination of AEE and alkaline pre-treatment resulting in the extraction of 90% of total lipid (Wu et al., 2017).

The most promising alternative method is SFE (Sookwong and Mahatheeranont, 2017; Xue et al., 2018). The main advantage of SFE is its ability to recover high purity FA product while using safe and nontoxic solvents (such as chemically inert supercritical fluids) making it a ‘greener’ alternative (Liu et al., 2017; Catchpole et al., 2018; Giacometti et al., 2018; Onumaegbu et al., 2018; Li et al., 2019). Previously, SFE using supercritical CO_2_ (SC-CO_2_) has demonstrated the extraction of ω-3 FAs from *Arthrospira* and is becoming a favourable FA extraction method for microalgae (Dibendetto, 2012; Li et al., 2019). Unlike traditional methods SFE requires dried biomass, with prior cell disruption and entails costly and highly specialised equipment. Various parameters such as temperature, pressure and CO_2_ flow can be successfully manipulated to improve the purity and yield (low-temperature SFE was observed to preserve quality/reduce processing) (Dibendetto, 2012; Leone et al., 2019). Previously with *Nannochloropsis* sp. maximum SFA, MUFA and PUFA yields using SFE (extraction yield of 94.28 mg/g and 18.39 mg/g of total lipid extracted) were achieved at 75°C, 550 bar and 14.48 g/min flow-rate, with maximum DHA (80%) obtained at 50°C, 550 bar and 14.48 g/min flow-rate (Leone et al., 2019). This corresponds to observations that increasing pressure improved PUFA concentration with DHA as the most available FA in the concentrate (29.3%–32.2%) (Dibendetto, 2012).

### 3.3 Non-disruptive pre-treatments

Prior to these extraction methods, additional non-disruptive pre-treatments may be necessary to break bonds between FAs and other compounds (i.e., remove attached phosphorous or carbohydrates) depending on the desired lipid (Cuellar-Bermudez et al., 2015). A strong example of this is the treatment of dried *Thraustochytrid* biomass with potassium hydroxide prior to solvent extraction, resulting in the recovery of 85% of total PUFAs (Onumaegbu et al., 2018; Kermanshahi pour et al., 2020). Cosolvents are also often introduced to the biomass mixture prior to improve solubility and aid during extraction. In some cases (in SFE especially with methanol as cosolvent), it has been observed to enrich PUFAs (such as DHA) in the final extract (Dibendetto, 2012; Kermanshahi pour et al., 2020). However, unlike disruption procedures these treatments are generally non-essential and are not widely considered to have as large an impact as either cell disruption or solvent choice (Breil et al., 2017; Khoo et al., 2020).

## 4 Cell disruption

**FIGURE 3:**
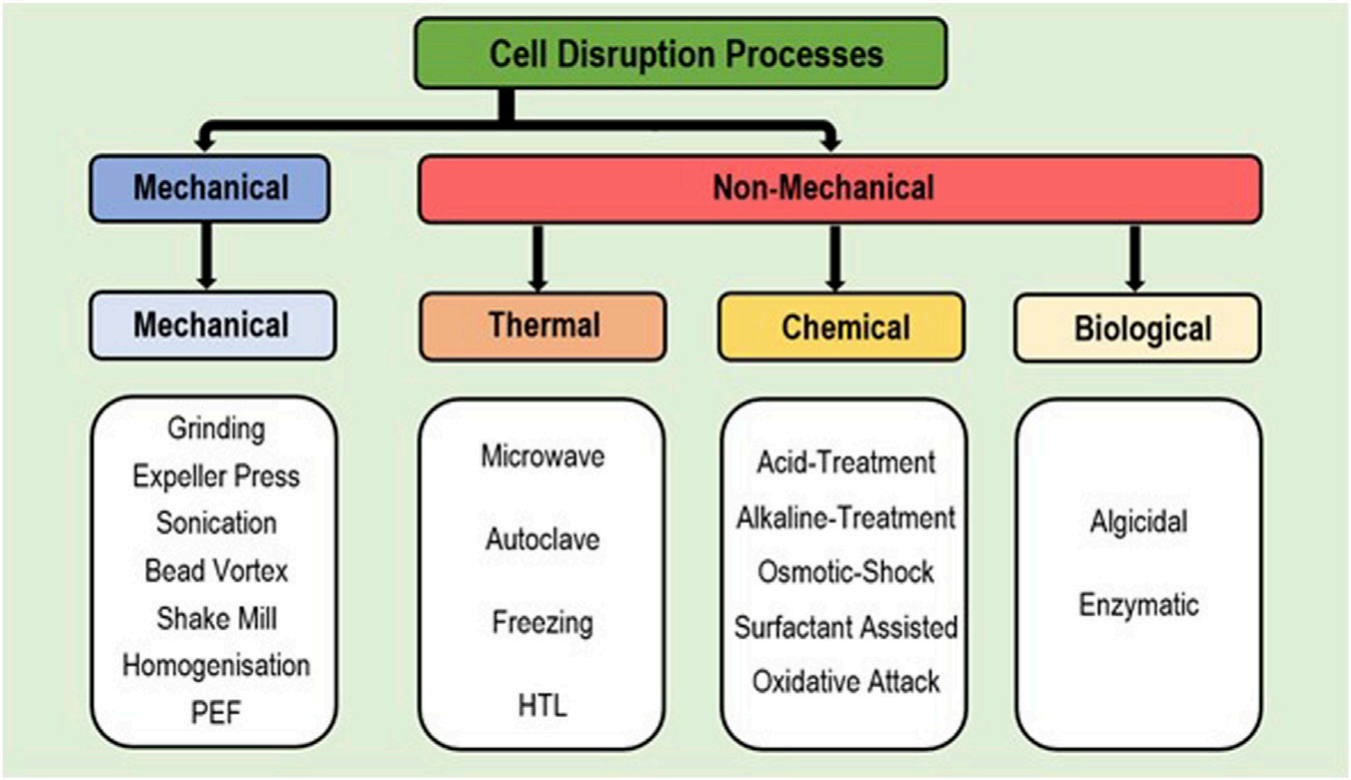
Flow-chart depicting the divisions of available cell disruption processes.

The use of extraction methods alone is often insufficient and can result in a significant loss of product (i.e., extraction of below 50% of cell lipids), due to thick cell walls blocking FA release or preventing the entry of specific solvents into the cell (Lee et al., 2012; Lee et al., 2017; Ramesh Kumar et al., 2019). Hence, cell disruption methods are frequently applied to better allow lipid release for solvent extraction (better access) for an improved recovery rate; with appropriate disruption key to high extraction efficiency (Prabakaran and Ravindran, 2011; Yellapu et al., 2018; Anto et al., 2020). Appropriate disruption is also essential for extraction from wet biomass due to reduced lipid transfer out of cells in the presence of high moisture (Howlader et al., 2018; Xue et al., 2018). While some extraction solvents naturally degrade the cell walls, alone this is insufficient to gain free access to full cellular content (Li et al., 2019). Disruption methods are divided into mechanical or non-mechanical, with non-mechanical further divided as chemical, thermal and biological (Figure 3 and Supplementary Table S1B) (Ramesh Kumar et al., 2019). Thermal and mechanical methods require high energy input while chemical methods. involve toxic and corrosive chemicals, and hence are generally not environment friendly (Lee et al., 2017; Zhang et al., 2018b; Khoo et al., 2020). Comparatively the use of enzymes to disrupt cell walls via biological extraction requires less energy input and needs no toxic chemicals; hence posing a far ‘greener’ option (Dibendetto, 2012; Giovannoni et al., 2020). Besides these major categories; use of pressurised gases (i.e., CO_2_, N_2_, N_2_O, or Ar) to disrupt cell walls has been recently explored as an emerging technique with wet biomass, which is favourable due to being cost effective and non-toxic in nature. However, further research is required to validate this as a viable process (Howlader et al., 2018). While disruption methods have generally been used independently, combination of multiple methods have enhanced FA extraction successfully (Kim et al., 2016).

### 4.1 Mechanical cell disruption

Mechanical methods of cell disruption are the most frequently used methods industry, often due to simplicity, ease of use, and general reliability (Dong et al., 2016; Kim et al., 2016). Yet many mechanical methods allow little increase in FAs obtained compared to standard manual grinding, with sonication, expeller press and shake mill exhibiting low impact on yield (Lewis et al., 2000; Byreddy et al., 2015). While sonication is simple and easy to scale-up, greater success has been achieved with the difficult to scale-up bead-vortexing procedures (Prabakaran and Ravindran, 2011; Byreddy et al., 2015). High-pressure homogenisation is more scalable but highly energy intensive, and requires specialist equipment (Lee et al., 2012; Hu et al., 2019). Both bead-vortexing and homogenisation use shear-force disruption, where force is directly applied to cells (Lee et al., 2017; Menegazzo and Fonseca, 2019). Similarly, pulsed electric field (PEF) treatment has been used successfully for cell disruption in microalgae (Khoo et al., 2020). However, all mechanical methods require high energy input and are thus not very eco-friendly, with specific techniques often difficult to scale-up (Lee et al., 2017; Hu et al., 2019; Nagappan et al., 2019). Hence, mechanical methods are often combined with non-mechanical methods to reduce energy consumption (Wang et al., 2015).

### 4.2 Chemical cell disruption

Chemical methods are less energy consuming than mechanical; however, they are highly selective to the cell-wall types which can be disrupted (Kim et al., 2016; Lee et al., 2017). Osmotic shock has high success for wet biomass disruption, and hence is an extremely favourable alternative (Enamala et al., 2018). Compared to mechanical treatment, osmotic shock may double yield, is more energy efficient, and uses a simple procedure (Byreddy et al., 2015). Alternatively, acid-catalysed disruption (using H_2_SO_4_) has exhibited relative success in releasing cellular lipids, particularly in improving wet biomass yields (Howlader et al., 2018). This method has been popular due to low cost and high efficacy, yet while acids are cheaper, they can be difficult to dispose of (require neutralisation) followed by numerous safety issues (Lee et al., 2017). This is similarly true for alkaline treatments (using NaOH) which degrades cell walls to a similar extent (Hu et al., 2019; Khoo et al., 2020). Furthermore, strong hydroxyl radicals have been used in disrupting cells for improved lipid yields in *Chlorella*; yet the catalysis procedure to create these free radicals is difficult and expensive to scale up (Kim et al., 2016; Sati et al., 2019). More recently surfactant-assisted extraction has gained attention whereby non-toxic surfactants are used to disrupt cell walls (Sati et al., 2019). This is ‘greener’ than most chemical methods due to the availability of biodegradable surfactants however, it is highly dependent on cell composition and thus requires further research into cost and efficiency (Howlader et al., 2018). In general, toxicity, cost and lipid degradation, limit the wider use of chemical methods; hence these chemicals must be recovered or re-used wherever possible (Kim et al., 2016; Lee et al., 2017).

### 4.3 Thermal cell disruption

Among the available thermal treatments, high success has been achieved via microwave techniques, especially in combination with traditional or enzymatic methods, resulting in improved oil quality and cell porosity (possibly by inhibiting lipid-degrading/modifying cell enzymes) (Martínez Herrera et al., 2019). Such treatments have shown improved yield over most mechanical techniques (sonication/ultrasound) and thus are viable alternatives (Kim et al., 2016; Liu et al., 2017). Microwave treatment has lower energy consumption than mechanical methods (but still higher than chemical and enzymatic), is a rapid procedure and is suitable for the combination of various solvents (Lee et al., 2017; Giacometti et al., 2018). Further, microwave efficiency may be enhanced by addition of salts such as, KCl, NaCl and CaCl_2_ (Li et al., 2019; Zeb et al., 2019). A simpler alternative to microwave is autoclaving of biomass, however commercial viability of this is low due to high energy consumption and potential lipid damage (Onumaegbu et al., 2018). Hence, biological methods are the most viable ‘green’ alternative disruption methods, which utilise enzymes to hydrolyse cell structures for lipid release (Cho et al., 2013; Martínez Herrera et al., 2019).

### 4.4 Biological cell disruption

Enzymatic degradation allows breakdown of cell walls and the release of cellular compounds without unnecessary energy input, chemicals or heat; being further desirable with enzymatic function able to be manipulated by modifying local conditions for additional optimisation (Cho et al., 2013; Sati et al., 2019; Khoo et al., 2020). However, enzyme selection is vital as due to varying cell wall constituents and not all enzymes can successfully degrade all microalgae and hence biological methods are the most selective (though due to similarities across Thraustochytrid species, enzymes can be suitable for multiple Thraustochytrid strains) (Byreddy et al., 2015; Enamala et al., 2018). Furthermore, reactions must be conducted under mild conditions due to enzyme sensitivity to their environment (Kim et al., 2016). Enzyme cocktails can be designed based on specific cell-wall constituents; with a broad range of enzymes trialled so far (Lee et al., 2017). Beta-glucosidase enzymes have shown great success with *C. vulgaris* (at pH 4.8 and 50°C), allowing solvent extraction yield to increase from 29.2% to 73.1% of total lipids (Cho et al., 2013). Similarly, cellulase, xylanase and pectinase have been compared for hydrolytic activity in *Scenedesmus* sp. and found that not only were specific conditions essential, but combinations were more highly effective than individuals (Zhang et al., 2018b). From this an enzymatic cocktail of cellulase, lipase and protease has been derived for a three-pronged attack on major cell structures (cellulose, lipid and protein) to achieve high FA recovery (88.3%) (Lee et al., 2017).

Other enzymes showing high potential for biological disruption include lysozymes, papain, trypsin, chitinases, amylases, mannase and pectinase depending on cell wall constituents (Jeevan Kumar et al., 2017; Howlader et al., 2018; Sati et al., 2019). Recently proteolytic enzymes in *Ananas cornosus* (pineapple) crude extract resulted in effective cell disruption without damaging lipids; more specifically bromelains in pineapple juice showed a 46.9% lipid yield (improved by combination with other methods) (Martínez Herrera et al., 2019; Rollins et al., 2022). Overall, this method poses a valuable exploration avenue for degradation by either individual enzymes, or combinations of multiple hydrolytic enzymes tailored to Thraustochytrids; with a ‘green’ procedure potentially developed from combining enzymatic methods with SFE being highly promising (Santos-Sánchez et al., 2016). Yet, current costs of certain enzymes in purified form, and lack of reusability, may limit the viability of enzymatic processes (Martínez Herrera et al., 2019). These methods are expensive and slow compared to thermal or mechanical methods but are significantly energy efficient and eco-friendly (Lee et al., 2017).

Algicidal treatments are considered a viable alternative biological treatment to enzymatic treatment (utilising micro-organisms that secrete algae-destroying compounds); yet the efficacy of such treatments generally relies on hydrolytic enzymes endogenous to the selected algicidal micro-organisms, and hence these may be described as a subset of enzymatic treatments (Wang et al., 2020). In this, algicidal agents (such as *Cytophaga*, *Flavobacterium* or *Alteromonas*) may be more applicable for industry scale production due to the ability of microorganisms to perpetuate and stay active whereas hydrolytic enzymes can lose catalysing abilities over time (Khoo et al., 2020). While the ability to grow micro-organisms producing their own hydrolytic enzymes is beneficial (cost-effective), these microbes can inhibit the growth of algal cells in coculture and may reduce biomass yields (Lee et al., 2017). Both algicidal and enzymatic methods are beneficial for wet biomass as they can easily be conducted in solution with high amounts of moisture (Howlader et al., 2018). Besides modifying cell disruption to improve yield, alternating extraction solvents used can also be a major determinant of efficiency and FA yield.

## 5 Extraction solvents for nutritional oils

For separation of FAs, a wide range of solvents have been trialled previously to optimise solvent extraction processes (Kumar et al., 2017). Many common solvents have significant issues, either relating to toxicity, availability or disposal, hence it is desirable to identify solvents that are efficient, non-toxic and environmentally friendly (Byreddy et al., 2015; Santos-Sánchez et al., 2016). Due to different species containing different lipid compositions, solvents cannot be generalised to all microalgae and thus require design based on the polarity of the lipid mixture present (Lee et al., 2012; Li et al., 2019). Often mixtures of polar and non-polar solvents are beneficial for lipid extraction (Kim et al., 2016). Hence, to determine ideal solvents, solubility of specific lipids must be considered along with the affinity of specific solvents to hydrophobic and hydrophilic portions of the lipid molecules (Meullemiestre et al., 2015; Li et al., 2019). Thus, an ideal solvent must allow high to very-high solubility of target compounds (Pätzold et al., 2019). Generally, long-chain FAs are low polarity, with non-water-miscible hydrocarbon solvents often used for extraction (Meullemiestre et al., 2015; Ramesh Kumar et al., 2019). Solvent mixtures may require a high degree of optimisation as extraction efficiency and solvent specificity impacts the lipid mixture composition and determine the downstream processing required (Kim et al., 2016).

### 5.1 Traditional extraction solvents

The most extensively used, well-known and effective solvent system is chloroform and methanol which is established in the standard Bligh/Dyer method (currently the preferred method) (Bligh and Dyer, 1959; Sati et al., 2019). However, this system has issues with toxicity and flammability, and is unfavourable for products intended for human consumption (Li et al., 2019). While chloroform is undesirable for mass use, no solvent equal in terms of efficiency has been identified thus far (Khoo et al., 2020). Ethanol/propanol systems are less toxic, but product recovery is poorer (Cuellar-Bermudez et al., 2015; Li et al., 2019). Such systems often require hexane use which has better recovery than ethanol/propanol but is again more toxic (Kumar et al., 2017). Hexane was found to be the most efficient individual hydrocarbon solvent (followed by chloroform), however a mixture of chloroform and methanol (used in Bligh/Dyer extraction) is more than twice as efficient, demonstrating the potential of combining multiple common solvents (biphasic system) (Byreddy et al., 2015; Yellapu et al., 2018). This may be insufficient alone, as it has been previously found that the order in which solvents are added also affects SFA, MUFA and PUFA profiles (Lewis et al., 2000; Cuellar-Bermudez et al., 2015). For example, extraction was observed to be 30% more efficient when multiple solvents were added in increasing polarity. (Lewis et al., 2000; Sati et al., 2019). Other widely used hydrocarbon solvents include; dichloromethane, diethyl-ether, toluene, heptane and isopropanol; but all are inferior to hexane or the combination of chloroform with methanol, and most have similar flammability and toxicity issues (Byreddy et al., 2015; Yellapu et al., 2018; Pätzold et al., 2019). Dimethyl carbonate (DMC) has recently been assessed as a ‘safer’ hydrocarbon due to its reduced toxicity, yet it is much more expensive (Kim et al., 2016). Thus, current research is now moving towards safer and more eco-friendly solvents than hydrocarbons.

### 5.2 Green extraction solvents

Several classes of alternative ‘green’ solvents with varying solving characteristics are currently under investigation including deep eutectic solvents (DES), ionic liquids (ILs), supercritical fluids, and terpenes (Kumar et al., 2017; Clarke et al., 2018; Zhang et al., 2018c; Pätzold et al., 2019). An ideal ‘green’ solvent is biodegradable, non-flammable, non-toxic, from renewable sources and has high extraction efficiency. DES in-particular are a highly promising solvent class as they can be designed from in-expensive renewable chemicals such as sugars, amino acids or choline chloride (vitamin B4) (Smith et al., 2014; Pätzold et al., 2019). These DES are defined as homogenous mixture of two separate compounds which has a reduced melting point compared to the individual components due the interaction between a hydrogen bond donor (HBD) and a hydrogen bond acceptor (HBA) (Smith et al., 2014; Pätzold et al., 2019). DES can be designed to be non-volatile, non-flammable, non-toxic, enzyme-compatible, and/or biodegradable depending on the constituent chemicals. Yet, some DES have high viscosity, which can impact the extraction, and thus-far they are relatively untrialled with ω-3 FAs (Pätzold et al., 2019).

ILs are defined as non-aqueous salt solutions maintained at temperatures between 0–140°C that have shown improved ω-3 FA recovery with dry and wet biomass (Kumar et al., 2017; Zhang et al., 2018c). Two frequently used ILs are imidazolium 1-ethyl-3-methylimadazolium ethyl-sulphate and tetra-butyl-phosphonium propanoate, which have shown extraction of 90% of lipids from dry biomass (Zhang et al., 2018c). Additionally, four further ILs (1-ethyl-3-methyl imidazolium acetate, 1-ethyl-3-methyl imidazolium diethylphosphate, 1-ethyl-3-methyl imidazolium tetrafluoroborate, and 1-ethyl-3-methyl imidazolium chloride) have shown efficacy comparable to traditional solvents (i.e., hexane or methanol) (Kim et al., 2016). While ILs can be recovered or recycled at the end of the process and are non-flammable, they are expensive and their toxicity to the environment requires further evaluation (although currently they are classed as low biotoxicity) (Kim et al., 2016; Zhang et al., 2018c; Howlader et al., 2018).

Terpenes are compounds derived from agricultural sources, that include D-limonene, P-cymene and A-pinene (Ciriminna et al., 2014; Kumar et al., 2017). These have been demonstrated to have significantly lower toxicity, and improved biodegradability and renewability compared to traditional solvents (Tanzi et al., 2012). While these terpenes are safer than standard hydrocarbons, D-limonene has shown FA recovery similar to hexane, making it a highly desirable alternative (Jeevan Kumar et al., 2017; Kumar et al., 2017). Supercritical fluids are used as organic solvents with SFE; with SC-CO_2_ (supercritical CO_2_) as the most common one (Elst et al., 2018; Yellapu et al., 2018). Supercritical fluids liquid at temperature and pressure above their critical point causing a change in thermo-physical properties and allowing use as ‘super solvents’ (Mishra et al., 1993; Dibendetto, 2012; Leone et al., 2019). A significant advantage is that SC-CO_2_ is considered a highly ‘green’ alternative solvent that can negate the evaporation step, as at room temperature CO_2_ is gaseous (Leone et al., 2019; Khoo et al., 2020). With all other solvents this evaporation step is essential and often requires additional equipment (a vacuum evaporator) and further energy input (Cho et al., 2013). A summary of solvent classes is provided in Table 1.

**TABLE 1:** Comparison of solvent classes for interchangeable use with lipid extraction procedures.

Solvent type	‘Greenness’	Benefits	Limitations	References
Standard Hydrocarbon (Met/Chl)	Low	⁃ Well-established	⁃ Highly toxic	Byreddy et al. (2015), Yellapu et al. (2018), Sati et al. (2019), Khoo et al. (2020)
		⁃ Very high extraction efficiency	⁃ Flammable	
		⁃ Good recovery rate	⁃ Hazardous to user	
		⁃ Large variety available	⁃ Difficult to dispose of safely	
		—	⁃ Generally require use of multiple solvents	
Non-standard Hydrocarbon (DMC)	Moderate	⁃ Lower toxicity than standard hydrocarbons	⁃ Very expensive	Kim et al. (2016)
		⁃ Similar efficacy to standard hydrocarbons		
DES	High	⁃ Comparatively more environmentally friendly	⁃ Untrialled with FA extraction from algae	Smith et al. (2014), Kumar et al. (2017), Khoo et al. (2020)
		⁃ Can be produced from simple and natural compounds	⁃ Few studies with lipids	
		⁃ High biodegradability	⁃ May require two or more DES for sufficient extraction	
		⁃ Non-toxic	⁃ Often require careful design on a per purpose basis	
		⁃ Non-volatile	—	
		⁃ Inexpensive	—	
Ionic Liquids	Moderate	⁃ Non-flammable	⁃ Very expensive	Kumar et al. (2017), Howlader et al. (2018), Hu et al. (2019), Anto et al. (2020)
		⁃ Thermal stability	⁃ Some IL’s harmful to humans	
		⁃ Suitable for wet or dry biomass	⁃ Few examples of use with microalgal lipids	
		⁃ No detectable vapour pressure	⁃ Further study required	
		⁃ Can allow single solvent extraction	—	
		⁃ Eco-friendly. Simple	—	
Terpenes (D-Limonene)	High	⁃ Low cost	⁃ Limited by supply of source material	Jeevan Kumar et al. (2017), Kumar et al. (2017), Patil et al. (2018), Sati et al. (2019)
		⁃ Can be used in Soxhlet extraction		
		⁃ Naturally derived		
		⁃ Environmentally friendly		
		⁃ Non-toxic		
		⁃ Suitable for wet biomass		
		⁃ Equivalent recovery to hydrocarbons		
Supercritical Fluid (Sc-CO_2_)	High	⁃ Low flammability	⁃ Difficult to optimise due to unpredictable impacts of temperature and pressure on supercritical fluids	Kumar et al. (2017), Liu et al. (2017), Sookwong and Mahatheeranont, 2017; Menegazzo and Fonseca (2019), Sati et al. (2019), Khoo et al. (2020)
		⁃ Non-toxic	⁃ Requires complex/specialised equipment which can be costly	
		⁃ Inert	⁃ Allows recycling of CO_2_	
		⁃ Environmentally friendly	⁃ Can only be used with SFE.	
		⁃ Removes evaporation step	—	
		⁃ Gives high extraction efficiency	—	
		⁃ Simple process	—	
		⁃ Potential to scale-up	—	
		⁃ Parameter flexibility	—	

## 6 Purification of fatty acids

### 6.1 Standard purification processes

As currently no solvents allow immediate isolation of individual FAs, purification steps are required to enrich the desired FA (DHA) in the extracted mixture. The enrichment of a specific ω-3 PUFA requires a multi-method process to achieve absolute purity. Traditional purification methods include fractional/molecular distillation, urea-complexation, low-temperature crystallisation and winterisation; with silver-nitrate-mediated complexation, enzyme-based methods and supercritical fluid chromatography (SFC) currently under investigation (Moharana et al., 2016; Li et al., 2019; Ramesh Kumar et al., 2019). The conditions and speed of purification is extremely important in preventing unwanted oxidation of PUFAs which can oxidise the extracted product (Cuellar-Bermudez et al., 2015). Fractional distillation is the most common method for separating PUFAs from the lipid mixture (Ramesh Kumar et al., 2019). This method utilises differences in molecular weights to allow separation and can be refined to molecular distillation which is even more selective but expensive (Namal Senanayake, 2010). In comparison, low-temperature crystallisation is straightforward, convenient and low cost but requires specialised equipment, uses large amounts of solvents and has low FA recovery (Li et al., 2019). This method uses different FA melting points to crystallise saturated and less-saturated FAs from highly unsaturated PUFAs (DHA melting point = −54°C) allowing selective enrichment (Namal Senanayake, 2010). Optimal purity requires several repeated fractionations and crystallisations, resulting in 91% DHA purity previously (Namal Senanayake, 2010). Urea complexation is also frequent due to low reagent cost, energy usage and rate of auto-oxidation (better PUFA preservation) (Li et al., 2019). In this method, SFAs and MUFAs are complexed with urea, which causes them to form crystals which are filtered out. This is fast, efficient, highly beneficial for PUFAs, and considered ‘green’ in using non-toxic chemicals and being useful as a ‘preliminary purification’ (Gunstone, 1996; Namal Senanayake, 2010).

### 6.2 Other purification processes

Enzymatic methods are beneficial as they do not require high energy input, large volumes of solvent, or cause damage to FAs. For example, phospholipase A1 (PLA1) was found to discriminate between ω-3 and non-ω-3 PUFAs during hydrolysis, allowing the breakdown of only SFA and MUFA and thus have potential for ω-3 concentration (Moharana et al., 2016). Alternatively, mixtures of lipases have been used successfully for hydrolysis to enrich desired PUFAs with over 79% DHA recovery observed. Furthermore, silver-nitrate-mediated chromatography has allowed isolation of up to 98% of DHA following pre-treatment with urea-complexation (Namal Senanayake, 2010). In this method temperature and pressure changes allowed optimisation of PUFA recovery. SFC shows high potential for PUFA separation, especially for EPA/DHA, with higher yield than alternative methods, and is an eco-friendly, non-toxic process (Li et al., 2019). It is important to note that a single purification method is insufficient and hence multiple methods must be used sequentially to enrich the desired FA.

## 7 Nanotechnology guided innovation in downstream processing of lipids

Thus far, minimal attention has been given to the possibility of using nanotechnological interventions for microalgal lipid separation. Nanotechnology is a significant and growing field in which extremely small artificial materials (featuring components generally between 100 nm and one atom in thickness) are chemically engineered to produce several unique and favourable attributes. These ‘nanomaterials’ have many beneficial characteristics (extreme strength, durability, flexibility, weight, precision, etc.) useful for materials developed to separate immiscible liquids or liquids of varying hydrophobicity (Gupta and Bowden, 2013; Gilmer et al., 2017; Wang et al., 2017; Yang et al., 2017; Zeng et al., 2017; Fu et al., 2018; Sun and Li, 2018; Liang et al., 2019).

### 7.1 Nanomaterials used in lipid separation

Previously few attempts have been made to separate lipid quantities using membranes with nanofiltration capabilities (Gupta and Bowden, 2013; Gilmer et al., 2017). While these materials have shown varying degrees of success, they have demonstrated that it is indeed possible to separate lipid fractions from a lipid mass using nanotechnology. The earliest study utilised a polydicylopentadiene (PDCPD) nanofiltration membrane in the separation of cis-fatty acids (CFAs) from SFA mixtures also containing trans-fatty acids (TFAs) (Gupta and Bowden, 2013). This was made possible due to the significant reduction in the permeation of CFAs through PDCPD in the presence of triisobutylamine. In addition, partial separation of different CFAs, such as OA and LA was achieved due to the membrane’s selectivity based on varying numbers of cis bonds present across different fatty acids (Gupta and Bowden, 2013). This study demonstrated the first industrially relevant possibility of high-throughput FA separation using nanomaterials and hence should be considered a significant pilot study in the application of nanomaterials in FA downstream processing. Recently 150 nm thick poly-epoxy nanofiltration membranes were generated with a high degree of crosslinking and have been demonstrated to offer size-based FA selectivity (using short, medium and long chain SFAs with 4, 11 and 18 carbon chains respectively) (Gilmer et al., 2017). It was found that altering the type and ratio of diepoxide and tripoxide monomers present in the crosslinking of the membrane allowed adaption and optimisation of selectivity towards different FA chain lengths, with production of membranes by spin-coating facilitating greater membrane flux. Selectivity of 21:1 was identified between short and long-chain FAs for membranes without optimisation (Gilmer et al., 2017). Subsequently, this was suggested to be highly applicable to improve FA separation in industry and provides further validation of the suitability of nanomaterials for the downstream processing of lipids. Nevertheless, this concept must now be taken further to consider a broader range of existing materials with potential applicability in FA separation.

### 7.2 Nanomaterials with lipid separating potential

Generally, nanomaterials identified to be potentially suitable for direct lipid separations (via nanofiltration) can be divided into two categories; nanomembranes (across which fluids can be selectively transported) and non-membranous nanomaterials (for compound separation without membrane crossing). An ideal nanomembrane for substance separation must have the qualities of strength/durability, selectivity and high permeability (for high flow rate/rapid processing of large volumes) (Zhang et al., 2018a; Sun and Li, 2018). In terms of general material separation (not specifically lipids), the potential application of paper-thin graphene oxide (GO) membranes for molecular sieving of substances has been highly considered as promising. (Yang et al., 2017; Liang et al., 2019). Such membranes may have thickness as low as 10 nm which is a key factor in the rapid permeation of liquids allowing quick ‘sieving’ (Sun and Li, 2018). Due to this fast permeation coupled with the material’s robustness, GO membranes may be suitable for nanofiltration, with highly laminated GO (HLGO) membranes successfully used for the filtration of organic solvents (Sun and Li, 2018). Compared to other nanomaterials, GO membrane synthesis is well-established and hence must be the first option considered for future investigation. Similarly, nanoporous atomically thin membranes (NATMs) have been suggested to allow selective transport of liquids but based on nanopore functionalisation to accept or reject molecules of different size or phobicity (i.e., exclusion by size or charge). (Wang et al., 2017). These NATMs contain rigid pores which are easily modified with functional groups allowing them to be ‘tuned’ to specific selectivity. Similar to GO membranes while theoretically suitable for separation of lipid from solvent or from different lipid, NATMs have not been tested experimentally for this purpose so far and thus require greater attention (Wang et al., 2017; Yang et al., 2017).

Multiple non-membranous nanomaterials have also been developed for the separation of crude/fuel oils from water; some of which have potential to be adapted/applied to consumable oils (such as nutritional oils). An interesting example is the SBS-SSM (superhydrophilic bump-stainless steel mesh) filter derived from *Stenocara* beetle shell structure (Zeng et al., 2017). In this, the bumps are superhydrophilic while the steel mesh is superhydrophobic/lipophilic allowing the separation of charged and non-charged liquids. This process has been shown to demulsify oil-water mixtures by capturing water droplets while allowing oil to permeate through the mesh (Zeng et al., 2017). This could be particularly useful following AEE extraction, which usually causes emulsion formation (thereby improving the viability of the process) (Kumar et al., 2017). Another non-membranous option is use of cupric-phosphate nanosheets which have been previously utilised in the separation of crude-oil from water (Zhang et al., 2018a). The advantages of these nanosheets are their chemical and thermal stability, and proven durability against contaminants such as salts, thus offering potential long-term re-usability. Unlike other nanotechnological options, this has been successfully tested with FAs intended for human consumption (soy-bean oil) along with its intended utilisation with fuel oils such as isooctane and diesel, demonstrating its strong potential (Zhang et al., 2018a).

Another alternative with the same intended function through different means is a delignified, polymer-modified wood bio-composite material. The basis of this as a ‘green’ nanomaterial is that wood is a cheap, durable and abundant material with low environmental impact (Fu et al., 2018). The bio-composite utilises the natural tiny endogenous channels in wood that can be delignified (via lignin removal) to provide a porous honeycomblike structure which is permeable to water. However, following treatment with a special epoxy mixture, the delignified wood rejects hydrophilic substances such as water while allowing the hydrophobic liquids such as oil to pass, facilitating selective absorption of oils (Fu et al., 2018). A particularly exciting aspect of this is that the modifications to the epoxy mixture were found to alter the charges accepted thus demonstrating a potential for the production of different bio-composite layers for separation of oils of different compositions. Both nanomembrane and non-membranous nanomaterial methods could be applied either to reduce/replace purification steps via separation of individual lipids or for removal of lipids from solvent mixtures (thereby replacing energetically unfavourable evaporation steps). The goals of using these materials are reduced energy consumption and usage of toxic substances, therefore, contributing to improving the overall ‘greenness’ of downstream processing.

### 7.3 Nanomaterials with alternative applications in downstream processing

In addition to direct lipid separation other areas of downstream processing such as cell disruption can be improved using nanomaterials. While biological cell disruption via enzymatic methods is among the most promising strategies for reducing energy consumption, there are some issues in using free enzyme, such as sufficient distribution and loss of activity (Martínez Herrera et al., 2019; Khoo et al., 2020). While this can be addressed by enzyme immobilisation in a solid material layer that has significant advantages, it is unsuitable for rapid processing of high volumes of cell biomass. Hence, the use of nanoparticles such as nanoporous core@shell particles (NPCSPs) can be used to immobilise enzymes and will allow greater freedom, mobility and contact with cells poses and interesting solution to this problem (Puri et al., 2013; Su et al., 2020). These nanoparticles have two layers, an inner shell and a core, with a large surface area and can be designed to adhere and support specific enzymes (i.e., a desired cocktail designed for disruption of algal cells) allowing controlled enzymatic reactions in solution (Su et al., 2020). This is valuable in allowing a high contact level between enzymes and cell walls while enzyme activity/integrity is better maintained. Furthermore, magnetic materials can be incorporated into the particles for easy recovery and reuse (Abraham et al., 2014). Thus, the consideration of enzyme application using NPCSPs may serve to make enzymatic cell disruption highly desirable.

## 8 Conclusions and future prospects

A complete ‘green’ process for SCO extraction from microalgae has not been established; hence significant development is required for its industrial adoption (Li et al., 2019). The processes requiring significant modification for improved ‘greenness’, cost reduction, yield and extraction speed include cell disruption, extraction methods, solvent types and purification. While current extraction methods (Soxhlet, Folch, Bligh/Dyer) are well established these must be further investigated to allow more efficient use of wet biomass and to function on a larger scale; with alternatives such as AEE and SFE to improve yields, reduce solvent or remove unnecessary steps (i.e., solvent evaporation) (Dibendetto, 2012; Kumar et al., 2017). A great concern in existing methods is the use of hydrocarbon solvents (i.e., hexane, chloroform or methanol) (Meullemiestre et al., 2015; Li et al., 2019). Thus, it is critical that substitutes such as SC-CO_2_, terpenes, ILs and DES are further investigated for their potential in reducing environmental impacts (Kumar et al., 2017; Pätzold et al., 2019).

Cell disruption is an essential step to enhance SCO yield from microalgal biomass. Current methods have high energy usage, cost, scalability issues or require undesirable chemicals (Prabakaran and Ravindran, 2011; Cho et al., 2013). However, osmotic shock and biological disruption methods such as hydrolysis offer improved yield with reduced energy input. In addition, PUFA purification strategies require significant optimisation to maximise DHA in the final product (but refinement processes well established/require no further work) (Gunstone, 1996; Cuellar-Bermudez et al., 2015). Hence, by developing existing processes, recovery of vital ω-3 FAs, such as DHA from Thraustochytrids can be significantly improved with less energy consumption and pollution and maximum product yield.

The production of dried biomass requires significant energy input and therefore development of extraction processes from wet biomass (Rollins et al., 2022) is vital to circumvent unnecessary energy usage. Thus-far there is no successful procedure that is non-toxic, produces low-no chemical waste, is energy efficient and has high PUFA recovery from wet biomass.

Finally, far greater consideration needs to be given to the role of nanomaterials by; 1) development of novel nanomaterials; and 2) the direct experimental testing of applicable nanomaterials for the improvement of fatty acid downstream processing. This is because nanomaterials offer a novel and highly unexplored avenue for the improvement of lipid separation; and with the enormous volume of already existing materials available, a large-scale study of multiple different materials with potential applicability to this purpose is both highly viable and desirable. This will thus eventually allow the separation of high-quality SCO and SCO-derived nutritional oils at an industrial scale using entirely sustainable sources and processes.
