# Supplementary material for: Development of sustainable downstream processing for nutritional oil production
Source: Front Bioeng Biotechnol. 2023 Oct 10;11:1227889. doi: 10.3389/fbioe.2023.1227889 (PMC10598382; doi:10.3389/fbioe.2023.1227889)
Supplement: Supplementary file 1 [file Table1.pdf]

Supplementary Table S1A: Comparison of standard and 'green' lipid extraction procedures

Extraction Procedure	'Greenness'	Solvent Requirement	Benefits	Limitations	References
**Folch**	LOW	Large amounts of organic hydrocarbons such as hexane, chloroform, methanol, etc. (may be possible to swap for terpenes)	▪ Allows management of large biomass samples.<br>▪ Straightforward process.<br>▪ No specialist equipment required.<br>▪ Potential to swap hydrocarbons for greener solvents such as DES, IL's and terpenes.	▪ Requires large quantities of solvent.<br>▪ Utilises toxic organic hydrocarbons.<br>▪ Can be laborious.<br>▪ Requires evaporation step.<br>▪ Does not remove toxins.	Folch et al. (1957), Meullemiestre et al. (2015), Enamala et al. (2018), Patel et al. (2018), Herrara et al. (2019), Khoo et al. (2020).
**Bligh-Dyer**	LOW (Improved if solvent altered)	Moderate amounts of organic hydrocarbons such as hexane, chloroform, methanol, etc. (may be possible to swap for terpenes)	▪ Solvents can be switched for greener alternatives.<br>▪ Simple and standard method.<br>▪ Easily modified.<br>▪ Potential to swap hydrocarbons for greener solvents such as DES, IL's and terpenes.<br>▪ Wastes less solvent than Folch.	▪ Usually utilises toxic organic hydrocarbons.<br>▪ Can be laborious.<br>▪ Unsuitable for large quantities of biomass.<br>▪ Requires evaporation step.<br>▪ Does not remove toxins.	Bligh and Dyer (1959), Meullemiestre et al. (2015), Enamala et al. (2018), Patel et al. (2018), Herrara et al. (2019).
**Soxhlet**	LOW (Improved if solvent altered)	Small amounts of organic hydrocarbons such as hexane, chloroform, methanol, etc. (may be possible to swap for terpenes)	▪ Solvents can be switched for greener alternatives.<br>▪ Allows continuous biomass processing.<br>▪ Solvent can be recycled repeatedly.<br>▪ Potential to swap hydrocarbons for greener solvents such as DES, IL's and terpenes.<br>▪ Less labour intensive.	▪ Usually utilises toxic organic hydrocarbon.<br>▪ Process is lengthy.<br>▪ Requires evaporation step.<br>▪ Does not remove toxins.<br>▪ Time consuming compared to Folch and Bligh-Dyer.<br>▪ Require correct apparatus.	Meullemiestre et al. (2015), Enamala et al. (2018), Patel et al. (2018), Herrara et al. (2019), Sati et al. (2019).
**Aqueous Enzymatic Extraction (AEE)**	HIGH	Water (or green solvents such as terpenes)	▪ Does not usually require extensive toxic solvents.	▪ Enzymes can be expensive.<br>▪Process is slow.	Du et al. (2017), Kumar et al. (2017a), Wu et al. (2017), Liu et al. (2019).

			▪ Highly useful for wet biomass. ▪ More energy efficient than standard processes. ▪ Cost-effective in reduction of solvent. ▪ Oil quality retained. ▪ Can remove toxins.	▪ De-mulsification step required (as emulsion forms). ▪ Functions poorly without prior disruption methods compared to other extractions methods.	
**Super-critical Fluid Extraction (SFE)**	HIGH	Supercritical fluids (Sc-CO_2)	▪ Highly efficient in lipid recovery. ▪ Independent of toxic hydrocarbon solvents and environment-friendly. ▪ Oil quality retained. ▪ Produces high purity products. ▪ Removes evaporation.	▪Requires careful optimisation of temperature and pressure. ▪ Functions poorly without prior disruption methods. ▪ Need costly and highly specialised equipment.	Elst et al. (2018), Giocometti et al. (2018), Xue et al. (2018), Anto et al. (2019), Kumar and Singh (2019), Leone et al. (2019), Menegazzo and Fonseca (2019)

Supplementary Table S1B: Comparison of major cell disruption methods for enhanced lipid extraction

Type	Method	Benefits	Limitations	References
Mechanical	Manual Grinding	▪ Can be used equally for all microalgae.<br>▪ Can be used to compare efficiency of alternative disruption methods.<br>▪ Low energy consumption.<br>▪ Simple and easy.	▪Time-consuming (very slow).<br>▪ Not scalable for use in industry.<br>▪ Usually used only as a comparative benchmark.	Byreddy et al. (2015), Enamala et al. (2018), Menegazzo and Fonseca (2019).
	Expeller Pressing	▪Can be used equally for all microalgae.<br>▪ Process is simple, easy and straightforward.<br>▪ Easily scaled up. Reduces solvents required.<br>▪ Fewer steps than alternatives.	▪Energy intensive. Slow and inefficient.<br>▪ Requires large biomass quantities.<br>▪ Unwanted heat production.<br>▪ Requires specialised machinery.<br>▪ Unsuitable for wet biomass.	Byreddy et al. (2015), Enamala et al. (2018), Patel et al. (2018), Menegazzo and Fonseca (2019).
	Sonication	▪ Reduced time.<br>▪ Can be used equally for all microalgae.<br>▪ High penetration into biomass. Improved extraction rate.<br>▪ Can reduce solvent required.	▪Energy intensive.<br>▪ Can form emulsions.<br>▪ Difficult to scale-up.<br>▪ Low improvement to accessibility.<br>▪ Small impact on lipid yield.<br>▪ Can damage product.<br>▪ Produces unwanted heat.	Enamala, et al. (2018), Onumaegbu et al. (2018), Kumar and Singh (2019), Sati et al. (2019), Khoo et al. (2020).
	Bead Vortexing	▪ Can be used equally for all microalgae.<br>▪ Can process large amounts of biomass.<br>▪ Highly scalable.<br>▪ High efficiency.<br>▪ Conditions can be manipulated.<br>▪ Can reduce required solvent.	▪ Energy intensive.<br>▪ Low improvement to accessibility.<br>▪ Beads must be separated for re-use.<br>▪ Produces unwanted heat by friction.<br>▪ Poor for thick cell walls/ rigid cells.<br>▪ Incomplete disruption.	Byreddy et al. (2015), Dong et al. (2016), Enamala et al. (2018), Khoo et al. (2020), Wang et al. (2020).
	Shake Mill	▪ Can be used equally for all microalgae.<br>▪ Reduced time.<br>▪ Simple process.<br>▪ Scalable.	▪ Energy intensive.<br>▪ Low improvement to accessibility.<br>▪ Small impact on lipid yield.<br>▪ Unwanted heat produced.<br>▪ Incomplete disruption.	Byreddy et al. (2015)
	Homogenisation	▪ Can be used equally for all microalgae.<br>▪ Very effective.<br>▪ Can process large amounts of biomass.<br>▪ Easy to scale-up.<br>▪ Good for high-moisture biomass.	▪Energy intensive.<br>▪ Low improvement to accessibility.<br>▪ Requires specialised equipment.<br>▪ Unwanted heat generation.	Dong et al. (2016), Howlader et al. (2018), Onumaegbu et al. (2018). Hu et al. (2019), Khoo et al. (2020).
	Pulsed-Electric Field (PEF)	▪Improves accessibility of cell contents.	▪Energy intensive.<br>▪ Impacted by media used.	Sheng et al. (2011), Patel et al. (2018),

		▪ Functions with wet biomass. ▪ Simple and fast.	▪ Potential destruction of compounds. ▪ High cost of maintenance.	Sati et al., (2019), Anto et al. (2020), Khoo et al. (2020).
Thermal	Microwave	▪ Improved oil quality. ▪ Fast and inexpensive. ▪ Non-contact heating. ▪ Improves recovery from wet biomass. ▪ Moderate potential to scale-up. ▪ Environmentally friendly. ▪ High extraction yield.	▪ Efficiency dependant on biomass type (Efficiency can be poor). ▪ Energy intensive. ▪ Not a standard commercial procedure. ▪ Set-up cost may be prohibitive. ▪ Increase temperature of mixture.	Kumar et al. (2017b), Howlader et al. (2018), Onumaegbu et al. (2018), Sati et al. (2019)
	Autoclave	▪ Can be used equally for all microalgae. ▪ Simple autoclaving of biomass. ▪ Allows high recovery. ▪ More efficient than mechanical methods.	▪ Energy intensive. ▪ May impact lipid structures. ▪ Less efficient than other thermal and enzymatic treatments.	Byreddy et al. (2015), Onumaegbu et al. (2018), Patel et al. (2018).
	Freezing	▪ Can be used equally for all microalgae. ▪ Easily coupled with other pre-treatments (especially mechanical).	▪Very energy intensive. ▪ Can be time consuming where repeated freezing thawing cycles are required (tedious process). ▪ Cell walls not completely disrupted.	Patel et al. (2018), Menegazzo and Fonseca (2019), Anto et al. (2020).
	Hydrothermal Liquefaction (HTL)	▪ Can use water as a major media. ▪ Efficient process. ▪ Useful for wet biomass recovery. ▪ Reduces need for solvents. ▪ Can be manipulated (acid/base/water).	▪ Energy intensive. ▪ Difficult to scale up to commercial scale. ▪ Very expensive. ▪ May utilise acids or alkaline solutions which can be difficult to dispose of.	Gollakota et al. (2018), Onumaegbu et al. (2018), Qu et al. (2018), Sati et al. (2019), Anto et al. (2020).
Chemical	Acid Treatment	▪ Low energy requirements. ▪ Can be used equally for all microalgae. ▪ Simple. ▪ Easily scaled up. ▪ No specialised equipment.	▪ Can damage extracted lipids. ▪ Requires neutralisation before disposal (impacts cost). ▪ More effective for dry biomass. ▪ Contaminate mixture requiring further separation step. ▪ Often requires mechanical agitation.	Dong et al. (2016), Onumaegbu et al. (2018), Zhang et al. (2018c), Hu et al. (2019), Sati et al. (2019).
	Alkali Treatment	▪ Low energy requirements. ▪ Can be used equally for all microalgae. ▪ Simple. ▪ Easily scaled up. ▪ No specialised equipment.	▪ Can damage extracted lipids. ▪ Requires neutralisation before disposal. (Impacts cost). ▪ More effective for dry biomass. ▪ Contaminate mixture requiring further separation step.	Dong et al. (2016), Onumaegbu et al. (2018), Zhang et al. (2018c), Hu et al. (2019), Sati et al. (2019).

			▪ Often requires mechanical agitation.	
	Osmotic Shock	▪ Low energy requirements.<br>▪ Simple.<br>▪ Easily scaled up.<br>▪ No specialised equipment.	▪ Time-consuming.<br>▪ Species dependant.<br>▪ Chemical recycling may be expensive.	Dong et al. (2016), Enamala et al. (2018), Onumaegbu et al. (2018), Howlader et al. (2018).
	Oxidative Attack	▪ Low energy requirements.<br>▪ Can be used equally for all microalgae.<br>▪ Potential to scale up.	▪ Can damage extracted lipids (By interfering in carbon chains).<br>▪ Relatively untested (very recent technique).	Kim et al. (2016), Sati et al. (2019).
	Surfactant Assisted	▪ Low energy requirements.<br>▪ Easily scaled up.<br>▪ Reduced toxicity/non-toxic.<br>▪ Can utilise biodegradables. Industrially viable.<br>▪ No specialised equipment.	▪ Species dependant.<br>▪ Requires further research.<br>▪ Needs study on limiting factors.<br>▪ Selection and recovery of surfactants difficult.	Kumar et al. (2017b), Zhang et al. (2018c), Menegazzo and Fonseca (2019), Sati et al. (2019).
Biological	Algicidal	▪ Low energy requirements.<br>▪ Can be further optimised by altering culture conditions.<br>▪ Safe for user.<br>▪ Generally, lysis is simple.<br>▪ Environmentally friendly.	▪ Can impede biomass growth.<br>▪ Species dependant.<br>▪ Need to carefully choose algicidal microbe used.<br>▪ Potential to impact amount of PUFA produced by algal cells.<br>▪ Media must be considered to facilitate both.	Lee et al. (2017), Howlader et al. (2018), Khoo et al. (2020), Wang et al. (2020).
	Enzymatic	▪ Low energy requirements.<br>▪ Can be further optimised by altering local conditions.<br>▪ Can be rapid.<br>▪ Non-toxic.<br>▪ Highly favourable for wet biomass.<br>▪ Safe for user.<br>▪ Highly efficient process.	▪ Can be costly depending on enzymes utilised.<br>▪ Highly species dependant.<br>▪ Difficult on industrial scale.<br>▪ Procedure not always simple.<br>▪ Needs stable conditions.<br>▪ Can be slow process.	Zhang et al. (2018c), Enamala et al. (2019), Herrara et al. (2019), Sati et al. (2019), Khoo et al. (2020), Wang et al. (2020).
